# Alpha-Lipoic Acid, Auraptene, and Particularly Their Combination Prevent the Metastasis of U87 Human Glioblastoma Cells

**DOI:** 10.1155/2023/8618575

**Published:** 2023-07-18

**Authors:** Azam Izadi, Mohammad Soukhtanloo, Farshad Mirzavi, Mohammad Jalili-Nik, Asie Sadeghi

**Affiliations:** ^1^Neuroscience Research Center, Institute of Neuropharmacology, Kerman University of Medical Sciences, Kerman, Iran; ^2^Pharmacological Research Center of Medicinal Plants, Mashhad University of Medical Sciences, Mashhad, Iran; ^3^Cardiovascular Diseases Research Center, Birjand University of Medical Sciences, Birjand, Iran; ^4^Department of Medical Biochemistry, Faculty of Medicine, Mashhad University of Medical Sciences, Mashhad, Iran; ^5^Department of Clinical Biochemistry, Faculty of Medicine, Kerman University of Medical Sciences, Kerman, Iran

## Abstract

**Background:**

The primary malignant brain tumor glioblastoma multiforme (GBM) is most commonly detected in individuals over 60 years old. The standard therapeutic approach for GBM is radiotherapy combined with temozolomide. Recently, herbal products, such as alpha-lipoic acid (ALA) and auraptene (AUR), have shown promising anticancer effects on various cancer cells and animal models. However, it is not well understood how ALA, AUR, and their combination in GBM work to combat cancer. Thus, the purpose of this study was to investigate the antimetastatic effects of the ALA-AUR combination on U87 human glioblastoma cells.

**Methods:**

The inhibitory effects of ALA, AUR, and the ALA/AUR combination on the migration and metastasis of U87 cells were evaluated using a wound healing test and gelatin zymography. The expression levels of matrix metalloproteinase MMP-2 and MMP-9 were assessed at the transcriptional and translational levels using quantitative real-time polymerase chain reaction (qRT-PCR) and western blotting, respectively.

**Results:**

Our findings revealed that combination therapy reduced cell migration and metastasis, which was indicated by the reduction in MMP-2/-9 expression both at mRNA and protein levels, as well as their enzymatic activity in U87 cells.

**Conclusion:**

This study demonstrated that the combination of ALA and AUR effectively inhibited the migration and metastasis of U87 cells. Thus, given their safety and favorable specifications, the combination of these drugs can be a promising candidate for GBM treatment as primary or adjuvant therapy.

## 1. Introduction

Glioblastoma multiforme (GBM) is a malignant and progressive brain tumor that arises from glial cells called astrocytes. GBM is classified into four stages based on the staging system developed by the World Health Organization (WHO) [1]. GBM mainly affects individuals over 60 years old and those with a family history of GBM and has a higher incidence rate in men compared to women [2]. The main molecular characteristics of GBM are the high proliferation rate and distant metastasis, which explain the low survival rates among GBM patients (14–16 months) [3]. Complete resection of the lesions followed by radiotherapy and chemotherapy with temozolomide is the current standard of care for managing GBM [4]. GBM tumors often grow quickly and recur either as a result of incomplete removal or resistance to radiation and chemotherapy [5, 6]. In recent decades, herbal and natural products have been highlighted as novel anticancer agents due to their specific properties [7, 8]. Therefore, in the current study, we examined the inhibition of the migration of glioblastoma cancer cells by the combination of alpha-lipoic acid (ALA) and auraptene (AUR).

### 1.1. ALA

**Figure 1:**
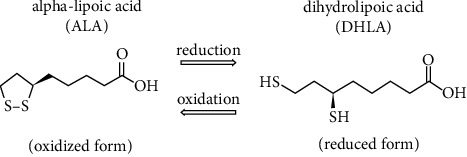
Molecular structure of alpha-lipoic acid and its reduced from dihydrolipoic acid (DHLA) [9].

ALA and its reduced form known as dihydrolipoic acid (DHLA) (Figure 1) are herb-derived compounds with notable antitumor effects [10]. Regarding its favorable properties and scant toxicity, ALA seems to be a desirable candidate for alternative therapy in cancer [11–13]. ALA is a pleiotropic compound with a variety of pharmacological activities, such as antidiabetic, antiobesity, anti-inflammatory, and hypotensive effects. ALA is also centrally involved in glutathione regeneration and vitamins C and E metabolism in addition to its ability to chelate heavy metals [14–17]. Furthermore, previous studies have shown that ALA effectively alleviates cancer cell proliferation and angiogenesis and stimulates the apoptosis of these cells; thus, it is considered a suitable candidate for cancer therapy [18, 19]. Recent studies have indicated that ALA attenuates cell proliferation by triggering the AMP-activated protein kinase (AMPK) signaling cascade and the consequent inhibition of the v-Akt murine thymoma viral oncogene/protein kinase-B (Akt/PKB) signaling [20, 21]. The proteolytic breakdown of the extracellular matrix components by gelatinases, such as matrix metalloproteinase MMP-2 and MMP-9, is a critical step in cancer development and metastasis [22, 23]. Previous studies have collectively suggested ALA as a potent inhibitor of MMP-2 and MMP-9 activities, which reduces the invasion of various cancer cells [24–26]. ALA also successfully induces cancer cell death by increasing the expression of proapoptotic genes such as Bcl-2-associated X protein (Bax) and decreasing the expression of the primary antiapoptotic gene, B-cell lymphoma 2 (Bcl2) [27, 28].

### 1.2. AUR

**Figure 2:**
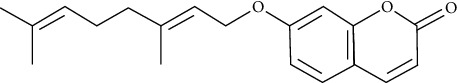
Molecular structure of auraptene.

AUR, also known as 7-geranyloxycoumarin, is a bioactive monoterpene coumarin ether (Figure 2) that is derived from the members of the *Rutaceae* and *Apiaceae* families [29]. AUR lacks toxicity and exhibits efficiency against various cancers; therefore, it can be proposed as a novel alternative anticancer agent [30, 31]. AUR is a pleiotropic compound with distinct pharmacological effects in the body including antidiabetic, antigenotoxic, anti-inflammatory, antioxidant, and immunomodulatory properties [32, 33]. AUR potently inhibits tumorigenesis and tumor proliferation by targeting various cytokines, growth factors, and transcription factors as well as their downstream signaling pathways. It also exerts regulatory effects on apoptosis and genes modulating cellular proliferation [34]. Consistently, AUR alleviates the proliferation of MCF-7 cells and suppresses cyclin D1, which is pivotal for cell cycle promotion and its dysregulation is widely observed in cancers [35, 36]. Similar to ALA, previous studies have proposed AUR to be a metastasis inhibitor indicated by the suppression of MMP-2 and MMP-9 enzymatic activities. Moreover, AUR efficiently decreased angiogenesis in several cancers by diminishing the expression of vascular endothelial growth factor (VEGFR)-1/2 [37–39]. In addition, AUR has been previously shown to suppress the Bcl-2 family expression and stimulate the Bax expression gene, thus acting as an apoptosis inducer in cancer [40, 41].

It is crucial to suggest new therapeutic techniques with higher success rates and fewer side effects because GBM is one of the most aggressive and malignant types of tumors and current medications occasionally fail or have undesirable side effects. In recent decades, herbal compounds have been widely used for cancer treatment due to their vast range of pharmacological effects, availability, and safety. Therefore, we examined the effects of the ALA/AUR combination on cell migration and metastasis of U87 cancer cells in the current study for the first time.

## 2. Materials and Methods

### 2.1. Materials

The cell line, human malignant glioblastoma (U87), was purchased from the Pasteur Institute (National Cell Bank of Iran), Tehran, Iran. AUR and ALA were obtained from Sigma-Aldrich (St Louis, MO, USA). Trypan blue, penicillin/streptomycin, dimethyl sulfoxide (DMSO), and trypsin-ethylenediaminetetraacetic acid (EDTA) were also purchased from Sigma-Aldrich (St. Louis, Mo, USA). High-glucose Dulbecco's modified Eagle's medium (DMEM) and fetal bovine serum (FBS) were procured from Gibco (Grand Island, NY, USA). The bicinchoninic acid (BCA) protein assay kit was purchased from Pierce Co. (Pierce, Rockford, IL, USA).

### 2.2. Cell Culture

The U87 cells were kept in a humidified incubator at 37°C and a 5% CO_2_ concentration. The cells were cultured in high-glucose DMEM supplemented with 10% FBS and 1% penicillin-streptomycin antibiotics, and the culture media were changed daily.

### 2.3. Scratch Wound-Healing Assay

The inhibitory effects of the ALA/AUR combination on the migratory properties of U87 cells were evaluated using the wound-healing scratch assay. A total of 7 × 10^5^ cells/well were seeded into 6-well plates. After 24 h of incubation at 37°C, when the confluency of the attached cells reached 90%, the cell monolayer was scratched using a sterile pipette tip. Then, the detached cells were cautiously washed with phosphate-buffered saline (PBS), and the attached cells were treated with 1/4 of the half-maximal inhibitory concentration (IC50) of each compound (AUR: 0.05 mM, ALA: 3.125 mM) and their combination. Images of the wound gap were captured using an inverted microscope at 0, 24, and 48 h posttreatment and analyzed with ImageJ software.

### 2.4. Gelatin Zymography

The enzymatic activity of MMP-2 and MMP-9 in U87 cells was determined using gelatin zymography [42]. For this purpose, U87 cells were treated with AUR (0, 0.2, and 0.4 mM), ALA (0 and 12.5 mM), and a combination of both (0.2 mM AUR-12.5 mM ALA). After 24 h of incubation, each sample's culture medium was collected and centrifuged at 13000 rpm at 4°C for 12 min, and the protein concentration of the supernatant was determined using the BCA technique. Equal amounts of the samples (20 *μ*g per lane) were loaded on a 10% separating SDS-PAGE gel with 0.1% gelatin. Every 20 min after electrophoresis, the gel was washed three times with washing buffer containing 2.5% Triton X-100 (to completely remove the SDS), and then it was kept at 37°C for 24 h in the developing buffer (containing 2.5% Triton X-100 in 50 mM Tris (PH 7.4), CaCl_2_ (5 mM), and ZnCl_2_ (1 *μ*M)). The gel was then stained with a staining buffer (0.5% Coomassie brilliant blue R-250 in distilled water containing 25% methanol and 10% acetic acid) at room temperature for 90 min. In the next step, the gel was destained in a 10% acetic acid solution until white bands appeared. The gelatinolytic activity of the samples (the density of the gelatine degradation zones) was captured by a GS-800™ calibrated densitometer (Bio-RAD, USA) and quantified using Image J 1.52a software.

### 2.5. Gene Expression Analysis

Using an RNA extraction kit (Pars Tous, Iran), the total RNA of the cells was extracted in order to further explore the expression of MMP-2 and MMP-9 after the ALA/AUR treatment of U87 cells. Then, using a NanoDrop spectrophotometer, the purity and quantity of RNAs were determined. In the next step, complementary DNAs (cDNAs) were created from RNAs by following the instructions of the Easy cDNA Synthesis Kit (Pars Tous Co., Iran). Quantitative real-time polymerase chain reaction (qRT-PCR) was conducted using SYBR Green PCR master mix (SMOBIO, Taiwan) on a Roche real-time thermal cycler.  Table 1 lists the specific primers for MMP-2 and MMP-9. Glyceraldehyde-3-phosphate dehydrogenase (GAPDH) was used as a housekeeping gene to normalize gene expression data, and fold changes were calculated using the 2^−∆∆CT^ method [43].

### 2.6. Western Blotting

For this purpose, 7 × 10^5^ U87 cells/well were seeded into a 6-well plate and incubated overnight. Then cells were treated with AUR (0 and 0.2 mM), ALA (0 and 12.5 mM), and ALA/AUR combination (0.2 mM AUR-12.5 mM ALA) for 24 h. Afterwards, radio-immunoprecipitation assay (RIPA) lysis buffer was used to lyse the cells, and the lysates were centrifuged. The obtained supernatants were kept at −70°C, and the BCA method was employed to measure the protein content of each sample, following procedures involving the separation of the proteins from the lysates using 7.5–15% SDS-PAGE and transferring them onto a polyvinylidene difluoride (PVDF) membrane. After skim milk blocking, the primary antibodies were diluted as instructed by the manufacturer (1 : 1,000 dilution), and then the membrane was incubated overnight with the primary antibodies at 4°C. In the next step, for 1 h, the membranes were treated with the horseradish peroxidase-conjugated secondary antibody (1 : 3000 diluted). The SuperSignal® West Femto kit (Thermo Fisher Scientific, Inc., USA) was used to detect the amounts of each protein. The relative expression was quantified using the Image J 1.52a software and was compared to that of the beta-actin protein as the housekeeping control [44].

### 2.7. Statistical Analysis

One-way analysis of variance (ANOVA) was used, followed by the Dunnett test, to compare the values and quantitative ratios obtained from the various groups. All experiments were run in triplicate, and the results are shown as mean (SD). *P* values less than 0.05 were considered to be statistically significant.

## 3. Results

### 3.1. AUR/ALA Combination Potently Inhibits the Migration of U87 Cells

**Figure 3:**
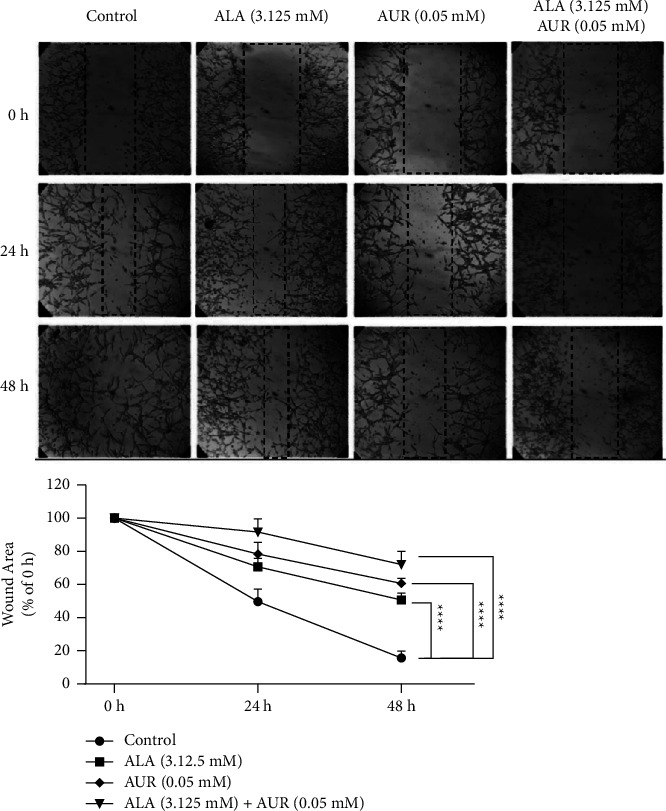
Comparison of the scratch area in the presence of ALA, AUR, and the ALA/AUR combination. Images of the wound area closure in U87 cells treated with 0.05 mM AUR, 3.125 mM ALA, or the AUR (0.05 mM)/ALA (3.125 mM) combination for 0, 24, and 48 h. Data are presented as the mean ± SD. ^*∗∗∗∗*^*p* value <0.0001 compared to the control group.

Tumor invasion and metastasis are only two examples of pathological and physiological processes in which cell migration plays a significant role, as indicated by numerous studies [45]. Thus, we performed a scratch assay to investigate the inhibitory effects of the ALA/AUR combination on cell migration. Our results are demonstrated in Figure 1. As observed, the ALA/AUR combination significantly diminished the migratory properties of U87 cells compared to monotherapy or the control group (Figure 3).

### 3.2. Gelatinase Activity Was Diminished by ALA/AUR Combination

**Figure 4:**
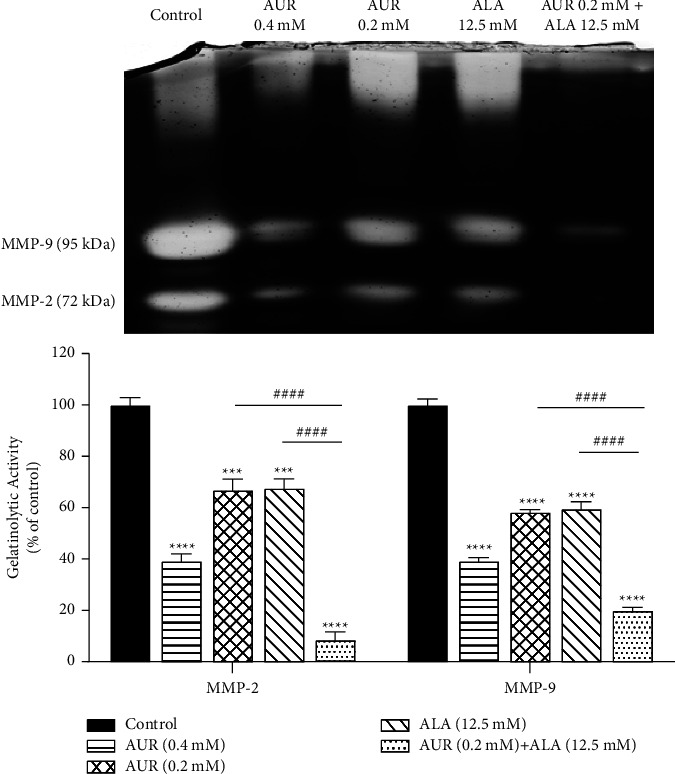
MMP-2 and MMP-9 gelatinase activities in the presence of ALA, AUR, and ALA/AUR. U87 cells were treated with 0.2 mM AUR, 0.4 mM AUR, 12.5 mM ALA, or the AUR (0.2 mM)/ALA (12.5 mM) combination for 24 h. The proteolytic activity of MMPs was analyzed using the gelatin zymography method. Data are presented as the mean ± SD. ^*∗∗∗*^*p* value <0.001, ^*∗∗∗∗*^*p* value <0.0001 compared to the control group, and ^####^*p* < 0.0001 compared to the ALA/AUR combination.

Here, we used gelatin zymography to examine the effect of the ALA/AUR combination on MMP-2 and MMP-9 enzymatic activities. In comparison with the control group, AUR and ALA, as well as the ALA/AUR combination, significantly reduced MMP-2/9 enzymatic activity in U87 cells. The combination significantly reduced MMP-2/9 activity compared to ALA and AUR monotherapy, as measured by the quantitative examination of gelatinolytic zones (Figure 4).

### 3.3. ALA/AUR Combination Decreased the mRNA Levels of MMP-2 and MMP-9

**Figure 5:**
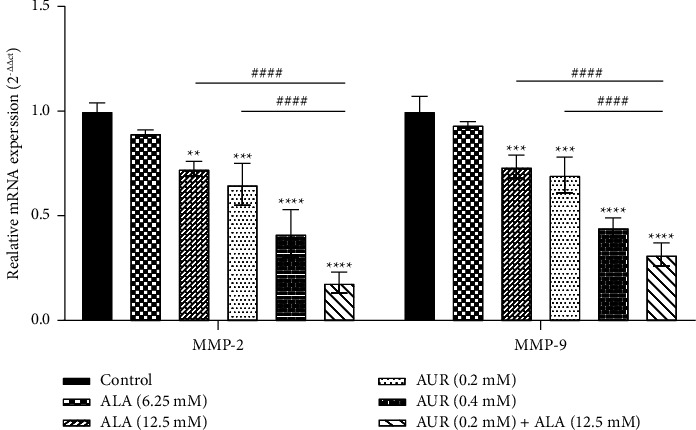
Evaluation of MMP-2 and MMP-9 mRNA expressions in the presence of ALA, AUR, and ALA/AUR. U87 cells were treated with AUR or ALA alone or the AUR/ALA combination for 24 h. MMP-2 and MMP-9 mRNA levels were analyzed using the qRT-PCR method. Data are presented as the mean ± SD. ^*∗∗*^*p* value <0.01, ^*∗∗∗*^*p* value <0.001, ^*∗∗∗∗*^*p* value <0.0001 compared to the control group, and ^####^*p* < 0.0001 compared to the ALA/AUR combination.

To further investigate the effect of ALA, AUR, and their combination on the transcriptional expression of gelatinases MMP-2 and MMP-9, the qRT-PCR method was employed.  Figure 5 indicates that the mRNA levels of MMP-2 and MMP-9 in U87 cells were significantly lowered after ALA, AUR, or ALA/AUR treatment compared to the control group. The ALA/AUR combination also exhibited a stronger inhibitory effect on MMP-2 and MMP-9 expressions compared to ALA or AUR monotherapy (*p* < 0.0001).

### 3.4. ALA/AUR Combination Attenuated the Protein Levels of MMP-2 and MMP-9

**Figure 6:**
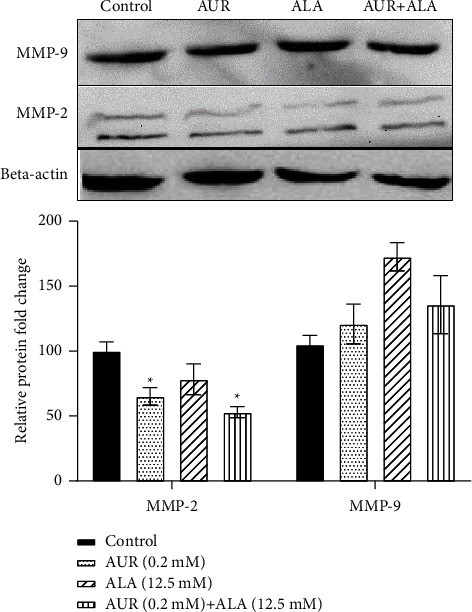
MMP-2 and MMP-9 protein levels in the presence of ALA, AUR, and ALA/AUR. U87 cells were treated with 0.2 mM AUR, 12.5 mM ALA, and the AUR (0.2 mM)/ALA (12.5 mM) combination for 24 h. The MMP-2 and MMP-9 protein levels were determined using the western blotting technique. The data are presented as mean ± SD. ^*∗*^*p* value <0.05 compared to the control group.

We employed western blotting to determine how the ALA/AUR combination affected the levels of MMP-2 and MMP-9 proteins in vitro. A significant reduction in MMP-2 levels was observed in the ALA/AUR combination group compared to the monotherapy or control groups (Figure 6).

## 4. Discussion

Tumor progression, which includes metastasis, is dependent on the ability to infiltrate adjacent tissues and spread throughout the body. The process of metastasis, which is responsible for malignancy and, as a result, the death of patients, is based on the migration and invasion of cancer cells [46]. Thus, this process has been particularly targeted for therapeutic interventions in numerous in vivo and in vitro studies. In the field of cancer treatment, some monotherapies may not be adequately effective, and, therefore, it seems necessary to boost the favorable effects of these therapies, limit the extent of drug resistance, and shorten the length of treatments. According to the previous studies based on cell and animal models, ALA and AUR have shown distinct anticancer effects as well as fewer side effects [34, 47]. However, no study has been conducted on the combination of both drugs regarding cell migration. Therefore, this study investigated the in vitro effects of the ALA/AUR combination on cell migration and metastasis of U87 cells for the first time. The obtained data demonstrated that the ALA/AUR combination exerts more antimigratory and antimetastatic effects than monotherapy with each of these compounds.

The effects of the ALA/AUR combination on cell migration were first assessed using a wound healing assay. In U87 cells, the ALA/AUR combination significantly suppressed cell migration relative to ALA and AUR monotherapies. Ji Jeon et al. reported that ALA inhibited cell migration and invasion by repressing the epithelial-mesenchymal transition (EMT) in thyroid cancer cells [21]. A study conducted by Yamasaki et al. revealed that ALA reduced cell migration through inhibitory effects on integrin *β*1 expression, which plays an important role in cell adhesion in bladder cancer cells [48]. The antimigration and antiangiogenic activities of AUR were also detected by Charmforoshan et al. and were found to be directly related to a reduction in cell migration and the mRNA expression levels of VEGFR-1 and VEGFR-2 [38]. Overall, ALA, AUR, and particularly the ALA/AUR combination can potentially be suitable candidates for hindering and controlling the metastasis of U87 cells.

Gelatinases, particularly MMP-2 and MMP-9, are efficient agents that trigger metastasis by causing the proteolytic destruction of extracellular matrix (ECM) components [22, 46]. These enzymes are released in their inactive form and are subsequently activated by a mechanism referred to as the cysteine switch. Numerous studies have demonstrated that MMPs are crucial to the metastatic process [47–49]. To investigate the activity of these enzymes, the gelatin zymography analysis was performed. The results revealed a remarkable inhibition of the gelatinase activity of U87 GBM cells after treatment with the ALA/AUR combination. In agreement with our results, it was reported that ALA suppresses the MMP-2 activity in mouse embryonic fibroblasts Balb/3T3 (3T3 cells) [49]. Furthermore, Lee et al. discovered that ALA significantly reduced the MMP-2/9 enzymatic activity while also suppressing metastasis and migration in the MDA-MB-231 breast cancer cell line [26]. Tripathy et al. highlighted ALA as an MMP-9 and TGF-*β* activity inhibitor, consequently attenuating the cell invasion and metastasis of 4T1 and MDA-MB-231 breast cancer cells [25]. Regarding the antimetastatic properties of AUR, Jamialahmadi et al. proved that AUR impedes the migration and metastasis of A2780 ovarian cancer cells by suppressing the MMP-2/9 activity [37]. In a model of dextran sulfate sodium (DSS)-induced ulcerative colitis, the DSS-induced increase in the enzymatic activity of MMP-2, -7, and -9 was diminished by AUR therapy [39]. Altogether, in addition to indicating the prominent contribution of MMP-2/9 to cell migration, these findings highlight the notable modulatory effects of ALA and AUR treatment, particularly in combination with each other, on these enzymes.

To further investigate the effect of the drug combination on gelatinases MMP-2/9 and whether their enhanced activity is due to increased protein and expression levels, MMP-2/9 mRNA and protein levels were determined. The results showed that monotherapy with ALA or AUR could lead to a reduction in transcriptional and translational expression levels of MMP-2 and MMP-9 while the combination of both drugs exhibited a more potent effect. In accordance with our results, it was reported that ALA decreases MMP-2/9 expression in breast cancer cells and vascular smooth muscle cells [24, 25], thereby reducing cell migration. Similarly, Kawabata et al. showed that AUR diminished the expression of MMPs in colorectal adenocarcinoma cells through the modulation of the extracellular signal-regulated kinase (ERK) signaling pathway [50]. In addition, it was reported that AUR reduced MMP-9 secretion in lipopolysaccharide (LPS)-stimulated human macrophages [51]. Therefore, it can be concluded that the combination of AUR/ALA decreases gelatinase activity partly via downregulating MMP-2/9 expression.

## 5. Conclusion

According to our results, the combination of ALA and AUR showed promising potential in the treatment of GBM by reducing the expression and activity of proteins involved in metastasis and migration, such as MMP-2 and MMP-9. This combination is safe and exhibits remarkable antimetastatic effects against GBM, but its efficacy should be verified in other cell lines and animal models. Furthermore, mechanism-based investigations are needed to fully elucidate the mechanism underlying the anticancer and antimetastatic effects of the ALA/AUR combination. Notably, there is still a dearth of data regarding the combination of these compounds, and the characterization and identification of the components are crucial to address all concerns; nonetheless, our data collectively suggest this combination as an alternative antimetastatic agent.

## Figures and Tables

**Table 1:** Sequences of qPCR primers.

Gene symbols	Primers (5′ ⟶ 3′)
MMP-9	Forward: GCATAAGGACGACGTGAATG
	Reverse: TGTGGTGGTGGTTGGAGG

MMP-2	Forward: TGGCAAGTACGGCTTCTGT
	Reverse: AGCTGTCATAGGATGTGCCC

GAPDH	Forward: ACAACTTTGGTATCGTGGAAGG
	Reverse: GCCATCACGCCACAGTTTC

## Data Availability

All data generated during this study are included within the article.

## Abbreviations

AUR: Auraptene
ALA: Alpha-lipoic acid
Bax: Bcl-2-associated X protein
Bcl2: B-cell lymphoma 2
EMT: Epithelial-mesenchymal transition
GBM: Glioblastoma multiforme
MMP: Matrix metalloproteinase.
